# Soluble interleukin‐6 receptor in young adults and its relationship with body composition and autonomic nervous system

**DOI:** 10.14814/phy2.14315

**Published:** 2019-12-23

**Authors:** Henry H. León‐Ariza, Daniel A. Botero‐Rosas, Edward J. Acero‐Mondragón, Dario Reyes‐Cruz

**Affiliations:** ^1^ Faculty of Medicine Doctorate in Biosciences PROSEIM Research Group University of La Sabana Chía Colombia; ^2^ Faculty of Medicine PROSEIM Research Group University of La Sabana Chía Colombia

**Keywords:** autonomic nervous system, heart rate, IL‐6 receptor, inflammation

## Abstract

**Background:**

The immune system generates inflammatory responses through cytokines like Interleukin 6 (IL‐6) and the Tumor Necrosis Factor alpha (TNF α); these cytokines mediate cellular responses aided by the presence of soluble receptors such as: Soluble Interleukin 6 Receptor (sIL6R) and Soluble Tumor Necrosis Factor Receptors Type 1 and 2 (sTNFR1, sTNFR2); the literature is limited about the relationship between this cytokines and the role of its soluble receptors.

**Objectives:**

This study is to determine a possible relationship between specific inflammatory markers and their soluble receptors with the autonomic nervous system's activity and body composition.

**Methods:**

27 subjects (13 men of 19.3 ± 1.6 years old and 14 women of 19.1 ± 1.7 years old) were evaluated. Body composition, autonomic nervous system activity and plasma concentration of inflammatory markers IL‐6, TNF α, sIL6R, sTNFR1 and sTNFR2 were measured using bio‐impedance, heart rate variability and ELISA respectively.

**Results:**

A positive association between body‐fat percentage and the sIL6R (0.47, *p* = .013) as well as inverse relationship between muscular mass and the sIL6R (−0.45, *p* = .019) were found. The sIL6R was also positively correlated with sympathetic activity markers: Relation LF/HF (0.52, *p* = .006), cardiac sympathetic index (0.45, *p* = .008), and cardiac vagal index (−0.44, *p* = .022).

**Conclusion:**

This study suggested that the IL‐6 trans‐signaling involving both the soluble receptor, sIL6R, and gp130 membrane co‐receptor could produce inflammatory responses that generate an impact on the autonomic nervous system, possibly due to its direct action on the hypothalamus, the solitary tract nucleus, or the heart.

## Graphical abstract

Studies on the immune system inflammatory responses are the focus on the cytokines like the interleukin 6, but there are not many studies that focus on the relationship between the soluble receptors and the impact on the autonomous nervous system, possibly due to its direct action on the hypothalamus, the solitary tract nucleus, or the heart.

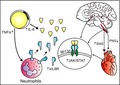

## 1 INTRODUCTION

The process of inflammation provides a protective response for tissues facing damage. It is dependent on the activation of the immune system, which generates responses in different tissues. The inflammatory response can be acute or chronic, depending on the increase in cytokines in plasma, most of which are produced by the immune system cells (Hotamisligil, 2006).

Some well‐known inflammatory markers are TNFα, IL‐6, C reactive protein, vascular cellular adhesion molecules type 1, Intercellular Adhesion Molecule 1, plasminogen activator inhibitor‐1, fibrinogen, P‐selectin, among others (Hummasti & Hotamisligil, 2010).

Substances like IL‐6 are considered pleiotropic, meaning that they have both pro‐inflammatory and anti‐inflammatory effects; however, that depends on whether the sIL6R, is present in plasma or not (Scheller, Chalaris, Schmidt‐Arras, & Rose‐John, 2011). It also depends on the presence of a co‐receptor, gp130, on the cellular membrane (Demyanets, Huber, & Wojta, 2012). The sIL6R is now being considered a more crucial inflammatory marker than the IL‐6. Furthermore, in the presence of sIL6R, the signaling is termed trans‐signaling; meanwhile, in its absence, it is named classic (Moreno Velásquez et al., 2015). Other inflammatory markers like TNFα also have soluble receptors (sTNFR1 and sTNFR2), which in chronic systemic inflammatory processes are elevated (Neirynck, Glorieux, Schepers, Verbeke, & Vanholder, 2015; Patel et al., 2016).

Although there is evidence that relates the behavior of different inflammatory markers with variations of autonomic activity evaluated using heart rate variability (HRV), this research focuses on cytokines like TNFα and IL‐6. The possible effect of the IL‐6 soluble receptors has been poorly studied, except for experiments done on rats that evidenced the presence of gp130 in the hypothalamus and circumventricular organs, areas that affect the autonomic nervous system responses (Schöbitz, Kloet, Sutanto, & Holsboer, 1993; Vallières & Rivest, 1997).

On the other hand, the relationship between high body‐fat percentage and inflammatory markers like TNFα, IL‐6, and sIL6R has been shown (Sindhu et al., 2015); however, the results in non‐obese people are inconclusive, and there is no evidence showing the relationship between muscle mass and sIL6R (Sindhu et al., 2015).

### 1.1 Objectives

The purpose of this study is to determine the relation between the concentration of inflammatory markers and their soluble receptors with the autonomic nervous system's activity, in addition to explore the relationship between inflammatory markers and body composition in this specific population.

## 2 METHODS

This study involved 27 Colombian university undergraduate students of medicine, from Bogota city (13 men of 19.3 ± 1.6 years old and 14 women of 19.1 ± 1.7 years old), who were chosen through convenience sampling and fulfilled the following criteria of inclusion: no consumption of prescription medicines, alcohol or cigarettes prior to and during the investigation as well as absence of pathological conditions including heart arrythmias, acute coronary events, diabetes mellitus, arterial hypertension, dysautnomic syndromes. Furthermore, those subjects fulfilling the inclusion criteria but 48 hr before the evaluation: consumed stimulant drinks such as coffee or tea, performed physical exercise or presented clinical manifestations of inflammatory processes, pain or infection, were excluded from the study.

### 2.1 Acquisition of the variables

#### 2.1.1 Body composition

It was obtained through bio‐impedance using a Full Body Sensor, Body Composition Monitor and Scale Model HBF‐510 (Omron), obtaining results of adipose tissue, the muscular mass, and visceral fat. The analysis was done first thing in the morning while the subject was fasting, not having done physical exercise, and after going to the bathroom.

#### 2.1.2 Autonomic nervous system activity

The HRV was used to evaluate the activity of the ANS. The resting heart rate was obtained through a beat to beat record (RR). Polar RS800CX heart rate monitor was used for 5 min in the supine position at an average temperature of 20°C, without the influence of air currents, noises, or lights (Sinnreich, Kark, Friedlander, Sapoznikov, & Luria, 1998).

### 2.2 Analysis of the variables

HRV analysis was done using the software Kubios VFC, University of Kuopio (Tarvainen, Niskanen, Lipponen, Ranta‐aho, & Karjalainen, 2014). During the pre‐processing of the heart rate signal, artifacts were removed (RR interval variations greater than 2.5 *SD* from the average), and the signal filtered using a high‐pass filter, Smoothness Priors, with a value of 300 Lambda and a cut‐off frequency of 0.035 Hz (Tarvainen et al., 2014).

From the RR tachogram, HRV time series was analyzed in the time domain: Average heart rate (HR), square root of the average of the differences between successive RR intervals (RMSSD), number of pairs of adjacent NN intervals differing by more than 50 ms in the entire recording (NN50) and the NN50 count divided by the total number of all NN intervals (pNN50) (Electrophysiology TF of the ES, 1996).

For the analysis in the frequency domain, the signal was interpolated at a rate of 4 Hz. Furthermore, through the Fast Fourier Transform, the spectral density and the power parameters were obtained (in total and percentage of total values) at very low frequency (VLF = 0 to 0.04 Hz), low frequency (LF = 0.04–0.15 Hz), and high frequency (HF = 0.15–0.4 Hz).

For non‐linear analysis, the Poincare Diagram was used to establish the *SD*1 and *SD*2 parameters. From these parameters, the CSI was calculated as the *SD*2/*SD*1 relationship, and the CVI was calculated as the log10 (*SD*1 × *SD*2) (Toichi, Sugiura, Murai, & Sengoku, 1997).

#### 2.2.1 Inflammatory activity

The blood samples were taken from the antecubital vein, in the morning while fasting and without any prior physical activity, consequently, IL‐6, Tumor Necrosis Factor‐alpha (TNFα), soluble receptor of Interleukin 6 (sIL6R), and the soluble receptors of the Tumor Necrosis Factor‐alpha type 1 (sTNFR1) and type 2 (sTNFR2), were measured by ELISA from this frozen plasma samples. The ELISA kits for the evaluation were provided by the commercial house (Abcam, Cambridge ‐ UK). All samples were analyzed in the same experiment to avoid errors in the intra‐assay variations.

### 2.3 Statistical analysis

The collected data were stored in a database in Excel 2016 (Microsoft Corporation), which was analyzed by the statistical program IBM SPSS Statistics for Windows, version 23.0 (IBM Corp., Armonk). In order to describe the variables, central tendency (mean) and dispersion with *SD* were measured. The homogeneity in the behavior of the variables was evaluated using the Levene statistic. An unpaired *t*‐test was used to establish the differences between men and women. Correlations between variables were established by the Pearson correlation coefficient establishing the relationship according to the value of *r* (There is no correlation *r* = 0.0–0.09; *r* = weak from 0.1 to 0.49, mean *r* = 0.5–0.74, significant *r* = 0.75–0.89, very strong *r* = 0.9–0.99 and perfect *r* = 1). The difference was considered statistically significant at *p* < .05 and highly significant at *p* < .01.

### 2.4 Ethical considerations

All participants started the evaluation process after signing the consent form. From the ethical point of view, the methods used are safe; thus, the research is classified as minimal risk research. According to Resolution 8430 of 1993, established by the Ministry of Health of the Republic of Colombia, all recorded information was strictly private and confidential. The ethics committee approved the protocol of the University of La Sabana. Additionally, all techniques were performed according to the Helsinki declaration of 1975 and all modifications about it.

## 3 RESULTS

When comparing men and women, significant differences in weight and height were found. Women presented a higher percentage of fat 30.5% ± 8.7% than 13.6% ± 3.1% in men, while men presented a higher percentage of muscle mass 49.8% ± 6.4% than 37.2% ± 4.6% in women. The resting heart rate was higher in women than in men, 80.4 ± 12.2 beats per minute compared to 66.9 ± 16.3 beats per minute *p* = .02, without significant differences in the parameters derived from HRV. The concentrations of inflammatory cytokines and their soluble receptors (IL‐6, sIL6R, TNFα, sTNFR1, and sTNFR2) were not statistically different between men and women (Table 1).

**Table 1:** General characteristics of the population

		Men *n* = 13 (*SD*)	women *n* = 14 (*SD*)	*p* value
General characteristics	Age (years)	19.3 (±1.6)	19.1 (±1.7)	0.798
	Weight (kg)	67.8 (±5.8)	58.9 (±7.2)	0.002^†^
	Height (cm)	174.1 (±5.5)	161.6 (±5.0)	0.0001^†^
	Fat (%)	13.6 (±3.1)	30.5 (±8.7)	0.0001^†^
	Muscle (%)	49.8 (±6.4)	37.2 (±4.6)	0.0001^†^
Heart rate variability	HR	66.9 (±16.3)	80.4 (±12.2)	0.024*
	RMSSD	81.7 (±45.1)	50.9 (±40.4)	0.069
	HFnu	0.54 (±0.15)	0.51 (±0.16)	0.616
	HF/LF	0.96 (±0.47)	1.19 (±0.89)	0.396
	CSI	1.88 (±0.72)	2.61 (±1.12)	0.052
	CVI	3.60 (±0.53)	3.29 (±0.49)	0.132
Inflammatory markers	IL‐6 (pg/ml)	2.5 (±1.2)	2.3 (±0.8)	0.616
	sIL6R (ng/ml)	67.1 (±12.4)	76.3 (±25.9)	0.549
	TNFα (pg/ml)	1.9 (±1.3)	2.1 (±1.3)	0.665
	sTNFR1 (pg/ml)	635.3 (±129.0)	564.6 (±88.8)	0.114
	sTNFR2 (pg/ml)	1858.5 (±311.5)	1932.2 (±360.9)	0.574

Note

Statistically significant *p < .05, ^†^ *p* < .01.

Abbreviations: %HF, Power of High Frequency expressed in percentage; CSI, cardiac sympathetic index; CVI, cardiac vagal index; HFnu, high frequency normalized units; HR, resting heart rate; LF/HF, low frequency high frequency ratio; RMSSD, the square root of the mean of the sum of the squares of differences between adjacent RR intervals;* SD*, standard deviation.

The relationship between cytokines and body composition showed different behavior in men and women (Table 2). The most representative relations were found in the sIL6R. In men, the concentration of sIL6R showed a directly proportional association with adipose tissue *r* = 0.57 and an inversely proportional association with muscle mass *r* = −0.61. In women, on the other hand, the concentration of sIL6R presented an inverse relationship with the muscle mass *r* = −0.32 and did not show any significant relationship with adipose tissue. After extensively analyzing men's and women's results alike, we found a positive association between the fat percentage and the sIL6R *r* = 0.47 emerged. Additionally, an inverse relationship with the muscular mass *r* = −0.45 was found.

**Table 2:** Relationship between cytokines and body composition organized by gender

		IL−6	TNFα	sTNFR1	sTNFR2	sIL6R
% Fat	All	−0.22	0.22	−0.19	0.22	0.47*
	Men	−0.36*	0.40*	0.42*	0.29	0.57^†^
	Women	−0.15	−0.12	−0.47*	0.07	0.04
% Muscle	All	−0.01	−0.16	0.13	−0.24	−0.45*
	Men	0.26	−0.44*	−0.48*	0.06	−0.61^†^
	Women	−0.34*	0.07	−0.03	−0.51*	−0.32*

Note

Statistically significant, *p < .05, ^†^ *p* < .01.

The results of inflammatory markers and HRV, evaluated by statistical methods in the time domain, were significant only for concentrations of sIL6R, demonstrating an inverse relation with RMSSD (*r* = −0.48; *p* = .012), NN50 (*r* = −0.54; *p* = .003) and pNN50 (*r* = −0.51; *p* = .007). The analysis in the frequency domain of HRV yielded a directly proportional relation between the sIL6R and the LF/HF ratio (*r* = 0.52 *p* = .006) and an inversely proportional relation with the high frequency activity. This is evaluated through the percentage of power (*r* = −0.48 *p* = .011) and activity of the high frequency normalized (*r* = −0.53; *p* = .005). The analysis of the nonlinear behavior of HRV relating to inflammatory markers showed a positive correlation between IL‐6 and the CSI (*r* = 0.39; *p* = .019) and between sIL6R and the CSI (*r* = 0.45; *p* = .008). On the other hand, we found negative relationships between: sTNFR2 and *SD*2 (*r* = −0.40; *p* = .038), sIL6R and *SD*1 (*r* = −0.48 *p* = .012), and sIL6R and the CVI (*r* = −0.44; *p* = .022) (Table 3).

**Table 3:** Relationship between cytokines and heart rate variability evaluated by different methods

		IL‐6	TNFα	sTNFR1	sTNFR2	sIL6R
HR	*r*	0.21	0.06	0.14	0.25	0.28
	*p*	0.294	0.778	0.496	0.211	0.162
RMSSD	*r*	−0.34	0.16	−0.28	−0.35	−0.48
	*p*	0.087	0.430	0.152	0.071	0.012*
NN50	*r*	−0.38	0.09	−0.26	−0.29	−0.54
	*p*	0.053	0.638	0.184	0.144	0.003^†^
pNN50	*r*	−0.33	0.03	−0.28	−0.32	−0.51
	*p*	0.093	0.871	0.163	0.104	0.007^†^
LF/HF	*r*	−0.01	0.00	−0.04	−0.12	0.52
	*p*	0.968	0.984	0.831	0.549	*0.006* ^†^
% HF	*r*	−0.16	0.17	−0.13	0.00	−0.48
	*p*	0.428	0.397	0.513	0.996	0.011*
HFun	*r*	−0.02	0.11	−0.16	−0.02	−0.53
	*p*	0.930	0.585	0.414	0.926	0.005^†^
*SD*1	*r*	−0.34	0.16	−0.28	−0.35	−0.48
	*p*	0.087	0.430	0.152	0.071	0.012*
*SD*2	*r*	−0.17	0.01	−0.35	−0.40	−0.32
	*p*	0.390	0.948	0.073	0.038*	0.104
CSI	*r*	0.39	−0.14	0.01	0.02	0.45
	*p*	0.019*	0.313	0.669	0.398	0.008^†^
CVI	*r*	−0.24	0.09	−0.33	−0.30	−0.44
	*p*	0.227	0.660	0.098	0.131	0.022*

Note

Statistically significant *p < .05, ^†^ *p* < .01.

Abbreviations: %HF, power of high frequency expressed in percentage; CVI, cardiac vagal index; HFnu, high frequency normalized units; HR, resting heart rate; LF/HF, low frequency high frequency ratio; NN50, Number of pairs of adjacent NN intervals differing by more than 50 ms in the entire recording; pNN50, NN50 count divided by the total number of all NN intervals; RMSSD, the square root of the mean of the sum of the squares of differences between adjacent RR intervals; SCI, cardiac sympathetic index; *SD*, standard deviation.

## 4 DISCUSSION

When body composition was evaluated alongside inflammatory markers, we found that in men and women overall, several inflammatory cytokines (TNF, sTNFR2, and sIL6R) manifested a positive relation with adipose tissue and a negative relation with muscle tissue. This result is consistent with publications that associate adipose tissue with inflammatory processes (Nishimura, Manabe, & Nagai, 2009), meanwhile linking skeletal muscle tissue with anti‐inflammatory responses (Gielen et al., 2003). However, the gender‐based differences regarding body composition suggest certain discrepancies in the differing inflammatory behaviors.

The analysis of inflammatory markers concerning ANS activity showed a poor relation, except for the sIL6R. It was associated in a statistically significant manner with sympathetic activity when analyzing the LF/HF ratio and CSI, and inversely with a parasympathetic activity using the RMSSD, the HF, and CVI. Previous studies have shown a relationship between inflammation and autonomic activity. However, until now, the relation had only been evident in well‐known inflammatory processes such as hypertension, diabetes, or cardiovascular disease (Cooper et al., 2015), but not in subclinical inflammatory processes (Kenney & Ganta, 2014).

None of the studies conducted involving the IL‐6 have demonstrated strong relation with adipose or muscle tissue. The findings were similar to the ones found in this study, which indicate that IL‐6 is a cytokine that is produced during pro‐inflammatory conditions, but at the same time, is produced by skeletal muscle tissue during physical exercise, which is an inherently anti‐inflammatory situation (Pal, Febbraio, & Whitham, 2014). Thus, evidence suggests that for the effect of being strictly inflammatory, it is required that trans‐signaling occurs (mediated by the soluble receptor IL‐6). Different from the classical signaling (receptor‐mediated membrane), which has, potentially, an energy regulatory effect and does not require the sIL6R (Scheller et al., 2011).

Studies with rats have identified that there is a high expression of gp130 in circumventricular sensory organs such as the terminalis vasculum organum laminae, which allows for the transition of inflammatory signals from the plasma to the CNS mediated by IL‐6/sIL6R (Vallières & Rivest, 1997) and in hypothalamic areas (dorsomedial nucleus, ventromedial and preoptic medial), where IL‐6 and its co‐receptor, gp130, are also evident (Schöbitz et al., 1993). This means that an increase in the plasmatic concentration of IL‐6 and sIL6R can stimulate hypothalamic areas, which are associated with responses such as fever or appetite reduction (Erta, Quintana, & Hidalgo, 2012).

The following has also been demonstrated in studies with rats: the effect of the IL‐6 trans‐signaling pathway on increased blood pressure when related to plasmatic changes in Angiotensin II (Coles et al., 2007), peripheral sympathetic responses generated by the intraventricular brain injection of IL‐6 that stimulates inflammatory processes on the CNS (Helwig, Craig, Fels, Blecha, & Kenney, 2008), and baroreceptor reflexes which reduce parasympathetic activity when IL‐6 is injected in nucleus of the solitary tract because of its prominent expression of gp130 in its cells (Takagishi et al., 2010).

Finally, cardiomyocytes generally express the gp130, like many other cells in the cardiovascular system (Hou et al., 2008), where the signaling mediated by IL‐6/sIL6R produced protective responses (anti‐apoptotic), but high concentrations promote cardiac hypertrophy, which is associated with the development of heart failure. The IL‐6 apparently has a direct effect on cardiomyocytes: reducing the chronotropic and inotropic response (Fontes, Rose, & Čiháková, 2015). Although the mechanisms are still not precise, they may involve processes that regulate channels or receptors associated with the JAK‐STAT signaling pathway in cardiomyocytes (Comini et al., 2005).

The results suggest continuing research on the potential effect of using the autonomic response as an inflammation marker in which the soluble receptors play a crucial role. The limitations of the study correspond to the small number of the sample of study participants. However, it opens a high possibility for future investigations.

## 5 CONCLUSION

This study suggested that the IL‐6 trans‐signaling involving both the soluble receptor, sIL6R, and gp130 membrane co‐receptor could produce inflammatory responses that generate an impact on the autonomic nervous system, possibly due to its direct action on the hypothalamus, the solitary tract nucleus, or the heart.

## CONFLICTS OF INTEREST

Participants declare no conflicts of interest.
